# The Effect of Different Soft Core/Hard Shell Ratios on the Coating Performance of Acrylic Copolymer Latexes

**DOI:** 10.3390/polym13203521

**Published:** 2021-10-13

**Authors:** Catalina Natalia Cheaburu-Yilmaz, Onur Yilmaz, Raluca Nicoleta Darie-Nita

**Affiliations:** 1 Academichem Kimya ARGE San. Tic. Ltd. Şti., Ege University Technology Development Zone, Bornova, 35100 Izmir, Turkey; catalina@academichemicals.com; 2 Physical Chemistry of Polymers Department, “Petru Poni” Institute of Macromolecular Chemistry, 41A Gr. Ghica Voda Alley, 700487 Iasi, Romania; darier@icmpp.ro; 3 Leather Engineering Department, Faculty of Engineering, Ege University, Bornova, 35100 Izmir, Turkey

**Keywords:** copolymers, core–shell, acrylate, latex, coating, leather finishing

## Abstract

Core–shell acrylic copolymer latexes containing bio resourced itaconic acid with different compositions in respect with the core and shell segments were synthesized, characterized, and applied as coating materials for leather. The purpose of the study was to evidence the high coating performance of the latexes when the ratio of the core/shell differed from 90/10 to 50/50 wt %. The copolymers were prepared via emulsion copolymerization technique and the products were isolated and characterized by means of structure identity, thermal behavior (DSC and DMTA), coating performance. The particle size of the latexes varied from 83 to 173 nm with the variation of the ratio of core/shell segments. The influence of the composition of soft part and hard part was highlighted in the thermal and coating properties. The optimal composition giving the best coating performance could be determined as DS 60/40. Further increase of the hard segment content, resulted in decreased emulsion stability and the coating performance on the leathers. The use of itaconic acid seemed to increase the emulsion stability as well the adhesion of the latexes to the substrate.

## 1. Introduction

Leather finishing is regarded as a stepwise process occurring in the final stage of leather manufacturing, and it is considered very important, as the final properties of leather goods such as appearance, coverage of surface defects and scratches, glossiness, feel, touch, color uniformity, fashionable effects, and patterns as well as mechanical and weathering resistance properties are defined. There are several factors that influence the finishing process, such as the characteristics of the leather, chemical products and formulation employed, and operating systems. However, the main factor effecting the appearance and performance of the leather surface is the coating composition. For this purpose, many categories of chemical products are used, i.e., pigments, waxes, binders, lacquers and other auxiliaries. Among these, acrylic latexes play the major role on leather properties, as they are the most widely used binders responsible for the film formation and binding the other additives to leather surfaces. In recent years, there have been many studies on improving the properties of leather coatings, as the demands for performance levels of leather surfaces are increasing [1,2,3,4,5,6,7,8,9,10,11,12,13,14,15].

It is known that the physical properties of latex polymer films depend closely on the macromolecular structure, the molecular weight, molecular inter- and intra-chemical crosslinking network structure, as well as the size and morphology of the latex particles [16,17]. Composite latex particles with well-defined particle morphology are used to improve the mechanical properties of polymers, to enhance adhesion properties and to modify the latex viscosity by varying the degree of neutralization [18]. These latexes usually offer better properties than conventional polymeric latexes and/or blending of two or more polymer components. These kinds of latexes are commonly known as core–shell or composite latexes and are usually made by two or multi-step emulsion polymerization where in the first step a seed latex (core) is formed using a monomer composition and in the second step another monomer composition is polymerized over the core to form the shell [19,20,21]. The composite polymeric latexes with two or more distinct phases may have various compositions that lead to different properties, such as high and low glass transition temperatures, hydrophobicity, reactivity, etc., which affect the properties of the final polymer. Several studies were reported on the preparation of composite latexes for various purposes [22,23,24,25,26,27,28,29,30,31,32,33,34]. Among these studies Limousin et al. [22] synthesized hybrid latex particles containing a hard, high T_g_ seed (styrene(S)/acrylamide (AM), T_g_ = 101 degrees C) and a low T_g_ (methyl methacrylate (MMA)/butyl acrylate (BA)/styrene(S), T_g_ = 13 degrees C) second phase polymer. They showed that the morphology of hybrid latex particles influenced the mechanical strength of polymer films. Lara et al. [26] also prepared core–shell latex-based adhesives to improve the adhesion of aluminum to poly (ethylene terephthalate) (PET) films and enhance the permeability of the final laminate to oxygen and water by using a soft acrylic component (the shell in core–shell particles) to improve adhesion, and occasionally a hydrophobic core to enhance the permeability. Zhang et al. [29] prepared PVAc-based inverted core/shell (ICS) structured latex adhesive with improved water- and heat-resistance by a copolymerizing-grafting sequential reaction approach for wood bonding. They reported that the wood bonding performance under heat and water conditions were effectively improved with core–shell morphology. Core–shell particles consisting of polybutyl acrylate (PBA) rubbery core and polymethyl methacrylate (PMMA)/polystyrene (PS) shell were synthesized via seeded emulsion polymerization by Roshanali et al. [34]. They also prepared silica loaded core–shell hybrid particles and examined the effect of addition of core–shell particles on mechanical properties of Bis-GMA/TEGDMA dental resins. They found that the composite containing 5 wt % Si-PBA-PMMA/PS particles showed 35% improvement in fracture toughness with respect to the neat matrix, without sacrificing flexural strength and flexural modulus.

The composite latex morphology can also be designed to obtain latex particles consisting of soft core and hard shell. The soft polymer may form a coherent film under ambient conditions, leading to a continuous film with a dispersed phase of hard polymer, which enhances the mechanical properties [35]. These types of latexes can be used, particularly, to improve the performance of water-based acrylic coatings to bring them closer to solvent based systems and/or polyurethane based coatings.

Itaconic acid (2-methylidenebutanedioic acid) is an unsaturated di-carbonic acid with broad application spectrum in the industrial production of resins and is used as a building block for acrylic plastics, acrylate latexes, super-absorbents, and anti-scaling agents [36]. Compared with citric acid the production of itaconic acid requires a cheaper technology, thus being considered a monomer from renewable resources. Liu et al. [37] reported poly(*n*-butyl acrylate)-poly(methyl methacrylate-itaconic acid) (PBA-*P*(MMA-ITA)) core–shell latex particles (CSR) synthesized via pre-emulsion and semi-continuous seeded emulsion polymerization process. The group studied the influence of itaconic acid content on the matrix toughness, having a soft core of poly(butylacrylate) and hard shell of poly(methyl methacrylate). The higher the content of itaconic acid, the tougher the polymer was. The COOH of itaconic acid seemed to modify the chain’s flexibility due to the extra hydrogen bonds formed between the polymeric chains. So, an amount of 6% would produce a tough polymer which is not always desired.

The present study refers to the synthesis and characterization of poly(ethylacrylate-*co*-itaconic-*co*-methyl methacrylate) copolymers with soft core of poly(ethylacrylate-*co*-itaconic acid) (PEA-*co*-IA) and poly(methyl methacrylate) (PMMA) as hard shell. The novelty of the present study is to contribute to the synthesis of composite latex binders with different ratios of soft/hard segments and to investigate their effect on coating performance in a specific industrial application. The study also constitutes a challenge of using itaconic acid as an acidic monomer unit in the composition to increase the functionality of the final polymer. Itaconic acid was added as a double carboxyl functional monomer in low portion to the monomer composition to contribute to particle stability of the emulsion, to increase the hydrogen bonding capacity and to give functionality for further crosslinking reactions to the final polymer. The expected outcome of the study could be to provide a route for the synthesis of soft core/hard shell latexes to optimize the performance and hardness levels of coatings especially used for soft surfaces.

## 2. Materials and Methods

### 2.1. Materials

Acrylic monomers such as methyl methacrylate (MMA, 99%), ethyl acrylate (EA, 99%) and itaconic acid (IA, 99%) were purchased from Sigma-Aldrich, Schnelldorf, Germany and used to synthesize the polymers via emulsion copolymerization. Disponil FES 77, sodium lauryl sulphate SLS 101 and Texapon P, products of BASF (Ludwigshafen, Germany) were used as co-emulsifiers special for the manufacture of finely dispersed and electrolyte–stable emulsions. Sodium bicarbonate (NaHCO_3_ > 99%, Sigma-Aldrich, Steinheim, Germany), ammonium persulfate (APS, >98%, Sigma-Aldrich, Steinheim, Germany) were used as reaction constituents as buffer agent and initiator, respectively. All chemicals were used as received without any further purification. Distilled water was used to as solvent to perform the reactions. The synthesis experiments were performed in a 4 necked 250 mL glass reactor equipped with a condenser, mechanical mixer, nitrogen inlet and dropping funnel.

### 2.2. Synthesis of Core–Shell Acrylic Copolymer Emulsions

Core–shell acrylic copolymers with a monomer content of 25 wt % were synthesized via two stage seeded emulsion polymerization technique as similarly described in previous papers [11,31]. Different compositions were set-up for the core–shell parts of the copolymers to evaluate their effect on the properties of final product and its application on leather as finishing material. For the synthesis, given amounts of emulsifiers and NaHCO_3_ were dissolved in water at room temperature and added in the reactor and mixed. IA, prior to be added inside the reactor, was dissolved in 70 °C water, then cooled down to room temperature. A total of 3.5 g of EA were added to the reactor and mixed to seed the reaction and form the pre-emulsion. The temperature was raised to 75 °C and APS was added to initiate the radical polymerization to form the seed. The reaction was continued by transferring the remaining EA monomer for 1h forming the soft core of copolymer. Subsequently, the hard shell phase was formed by transferring MMA for 30–45 min. Parallel, APS was dropped inside the system to provide new radicals for two hours. After all the transfers were completed, the system was maintained for another 2 h to complete the reaction. The conversion of the reaction was gravimetrically verified periodically, and final conversions were obtained over 99%. After cooling down the system particle free and blue-like emulsions were obtained, except for the DS 50/50 composition where some coagulum was formed. The final emulsions with a pH around 2 were neutralized to pH 5–6 with 30 wt % NaOH solution to be compatible with the other finishing constituents and pH of the leather. Table 1 shows the details of the experiments. Syntheses were performed in two parallels for each sample.

### 2.3. Characterization of the Copolymers

The synthesized polymers were characterized in terms of their particle size and polydispersity index by using a NanoZS zetasizer instrument (Malvern Instruments, Worcestershire, UK). The particle size measurements were performed with three parallel samples prepared for each latex and average values have been presented.

The structure was confirmed by FT-IR spectra of the copolymer films which were recorded with a Bruker VERTEX 70 spectrometer (Bruker, Billerica, MA, USA) by scanning in the range of 600–4000 cm^−1^.

Thermal properties of the polymeric films were tested via Differential scanning calorimetry (DSC) and dynamic mechanical thermal analysis (DMTA). The DSC thermographs were recorded with a Differential Scanning Calorimeter Shimadzu 60 Plus (Kyoto, Japan) at a heating rate of 10 °C/min under N_2_ atmosphere from −70 to 250 °C. Dynamic mechanical measurements were carried out using an Anton Paar MCR 301 Dynamic Mechanical Analyzer (Graz, Austria) equipped with CTD450, in the temperature range from 60 to 110 °C, under N_2_ atmosphere, with a heating rate of 3 °C/min, at 1 Hz frequency. All measurements were performed in extension mode and the storage modulus E′, the loss modulus E″, and the value of the tan δ (tan δ = E″/E′) were determined for all temperature range.

### 2.4. Application of Latexes in Leather Finishing

The synthesized core–shell polymer emulsions were used as pigment binders in leather finishing application to evaluate the effect of their composition on coating performance. The finishing mixture formulation was given in Table 2. Crust upper leathers were cut into (40 × 40 cm squares and used for finishing trials. The application of finishing mixtures was done via spray coating followed by drying and hot plating at given intervals. The finishing performance of the emulsion was evaluated by standard methods performed on finished leathers, such as Flexing endurance (ISO 5402–1:2011); color fastness of leather to To and Fro rubbing (ISO 11640:2012); color fastness to water spotting (ISO 15700:1998). The evaluation of all the tests related to color change was done according to the Grey Scale Standard method (IUF 131–132) and following the standard ISO 105 A02: 1993/ISO 105 A03: 1993 which provides a rating between 1 and 5 (5: means no color change, and 1: means failure).

## 3. Results and Discussion

### 3.1. Particle Size Analysis

The average values of the particle size and polydispersity index (PDI) values of the latexes were presented in Table 3.

**Figure 1:**
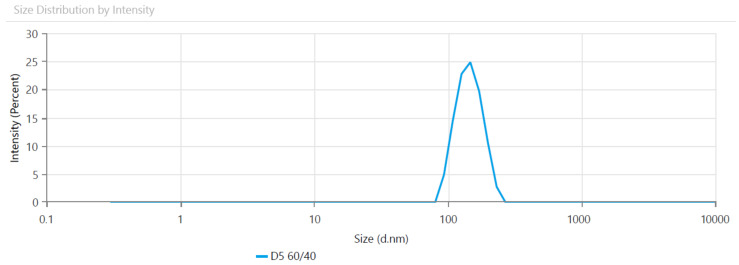
Size distribution as function of peak’s intensity for the DS 60/40 latex.

The particle size of the latexes varied from 83 to 173 nm in terms of copolymer composition. The results showed that an increasing average size of particles was obtained with the increase of hard–shell ratio, except DS 75/25. This can be due to the higher water solubility of MMA monomer and faster polymerization rate that decreases the control on the particles. However, to understand the phenomenon better further investigations on the correlation of particle size and MMA content is needed. On the other hand, the particle size and the PDI values were found to be low, promoting good pigment binding ability for coating applications. Other authors have also obtained controlled particle growth and a small risk of coagulation for latex particles obtained by seeded emulsion polymerization, the synthesis method used in the present study [38]. Moreover, itaconic acid with a double carboxyl functional side groups contributes to the anionic stability of the final emulsions. Figure 1 is an example of the particle size distribution of latexes.

### 3.2. FTIR Analysis

**Figure 2:**
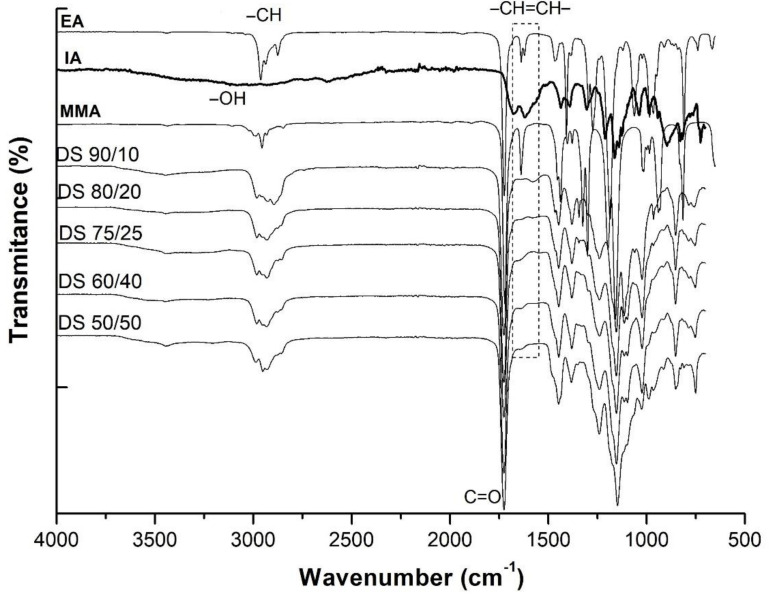
ATR–FTIR spectra of polymers as films and their pure monomer.

The structural identity was evaluated and confirmed by the ATR–FTIR spectra of the monomers and their polymers by comparison, as presented in Figure 2.

The FTIR spectra qualitatively proved the monomers conversion into the polymers by the disappearance of the band assigned to the vinyl groups in the polymers’ spectra (from 1640 cm^−1^). Figure 2 shows the main absorbance peaks of the copolymers observed at 2800–3000 cm^−1^ due to the –CH stretching, at 1730 cm^−1^ C=O stretching vibrations, at 1450–1386 cm^−1^ –CH_2_, –CH_3_ and –CH deformation stretching, at 1160 cm^−1^ stretching vibration of ester groups (O–C–). The characteristic peaks of the itaconic acid due to the stretching vibrations of –OH at 3000–3500 cm^−1^, –OH deformation at 1440–1390 cm^−1^, –C–O– at 1320 cm^−1^ and R–OH groups at 750 cm^−1^ were identified on the polymers’ spectra confirming the success of the copolymerization between the selected monomers as similarly observed by Loginova et al. [39].

### 3.3. Mechanical and Thermal Analyses

**Figure 3:**
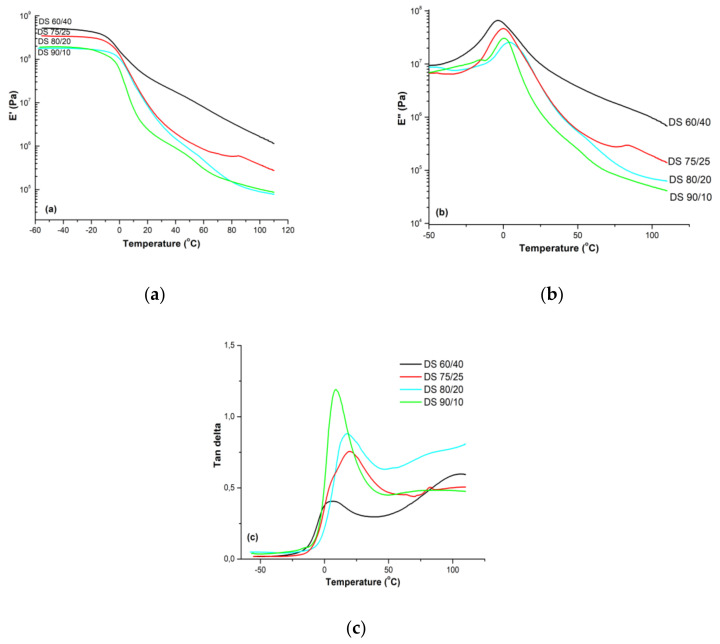
DMTA curves of the copolymer films function of temperature. (**a**) Storage modulus E′ (**b**) Loss modulus E″ (**c**) Tan δ.

Dynamical Mechanical Thermal Analysis (DMTA) was performed to assess the thermomechanical behavior of the latex films with different core/shell ratios. The analysis on latex film DS 50/50 could not be performed due to its brittleness as the test requires films to be deformed. The storage modulus (E′), loss modulus (E″) and tan δ’s values as a function of temperature were plotted in Figure 3 for the other latex films. From the results it can be seen that the plateau of storage moduli dropped sharply at the glass transition temperature (T_g_). Another observation was that as the hard–shell ratio increased, the storage moduli of the films increased proportionally. The latex film DS 60/40 showed much higher storage modulus values than others, while the lowest values were obtained for DS 90/10 as expected. By decreasing the amount of elastomeric EA, the modification of E′ was induced by a gradual change in the elastic contribution, therefore the stiffness of the material increased. The E′ curves continuously decreased with increasing of testing temperature as the latexes start softening. Very similar trends were also observed for loss moduli of the films. Tan δ can provide information on the overall flexibility of studied materials and the interactions between the components. The tan δ maximum peaks corresponding to the synthesized latexes were close to their E″ peaks and points where E′ began to drop, similar situation was reported by Zhang et al. for copolymers of poly (butyl acrylate) (PBA) and poly (ethyl acrylate) (PEA) with isoprene (IP) [40]. By following the evolution of tan δ values of latex films function of temperature (Figure 3c), an asymmetric double–peak structure can be observed mainly for DS 60/40 and slightly for DS 70/30 and DS 80/20, with a maximum at lower temperature and a shoulder at higher temperature. Core–shell polymeric materials with high shell T_g_ and low core T_g_ compositions have been produced also for medical applications in order to synthesize core–shell polymers that could be triggered by heat [41]. Thus, the shell can be selectively weakened by increasing temperature, while leaving the core exposed with the aim to release its encapsulations at a specific moment in a given targeted area.

The peaks observed within 0–25 °C temperature range exhibited the glass transition of the soft–core phase. However, shifts of the T_g_ to higher temperatures were obtained for all samples related to theoretical values which were estimated by using the Flory–Fox equation, a simple empirical formula that relates molecular weight to the glass transition temperature of a polymer system. Moreover, differences between the peak temperatures and their intensities were also observed for core–shell latex films, as similarly reported by Xu et al. [42]. The decrease in peak intensity was proportional to the increase in hard-shell ratio as expected. On the other hand, the differences in peak temperatures may possibly be due to different interactions between soft and hard segments. From Figure 3c it could be also observed that tan δ values showed an upward trend after the first transition due to the second transition of hard–shell phase, however, could not reach to the transition until the end of analysis temperature.

The area under the tan δ curve reduces with decreasing the amount of soft core (from DS 90/10 to 60/40), indicating less molecular mobility. Therefore, it could be mentioned that improved damping properties, meaning that the material can better absorb and dissipate energy, were recorded for latexes with higher amount of soft core. This finding agreed with the flexing endurance results, as discussed below in leather application section.

**Figure 4:**
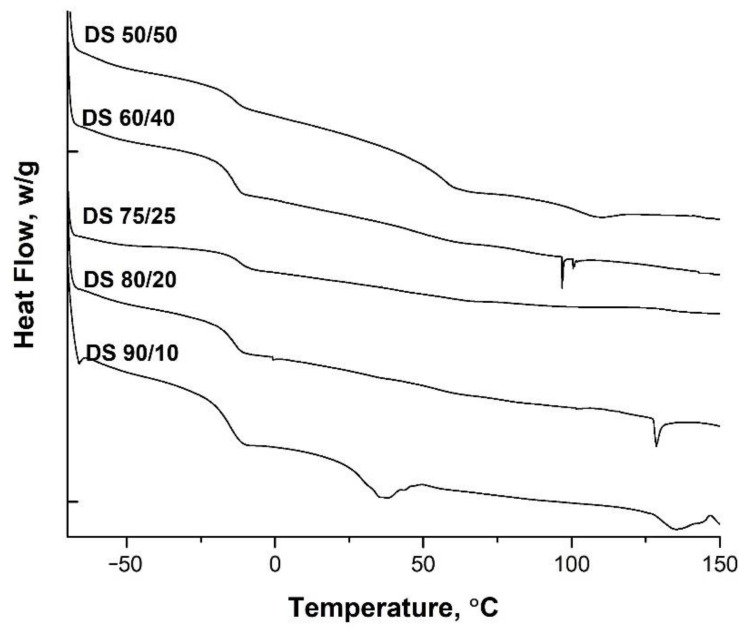
DSC curves of the acrylic copolymers with different compositions.

The DSC curves of the latex films were plotted in Figure 4. T_g_ of the films were depicted from the thermograms. The glass transitions of soft-core phase were clear and the T_g_ values were found to be between 16 and 12 °C for all samples which were close to theoretical value of −20 °C of poly(ethyl acrylate). Very close values of transition temperature for PEA within acrylic copolymers were found such as −18 °C by Zhang et al. [40], −21 °C [43] and −24 °C by Fytas et al. [44].

From the thermograms the transitions of the harder domains could also be observed. For the samples DS 90/10 and DS 50/50 the transitions were clear and found to be 31 and 57 °C, respectively. For the other copolymers the hard phase transition was small and observed near 50 °C. This kind of variation in transitions can occur due to the interactions at interfaces between the layers and may form intermediate interlayer phases as observed by Mu et al. [45]. The same outcome was also reported by Liu et al. [37], where the transition temperature was shifted between the values of the two components of the core–shell. In particular, they used itaconic acid in the shell part and observed that the functional monomer itaconic acid with polar group participated in the copolymerization reaction. The interaction between polar groups (–COOH) from itaconic acid’s structural unit enhanced the interaction force between the macromolecular chains, even the *P* (MMA–ITA) copolymer produced a slight physical cross-linking due to the intermolecular force and the hydrogen bond.

### 3.4. Leather Application

The performance results of the finished leathers are summarized in Table 4. The flexing endurance test was performed to assess the resistance of the finishing layer to stretching and pressing actions such as shoes exposed during walking.

**Figure 5:**
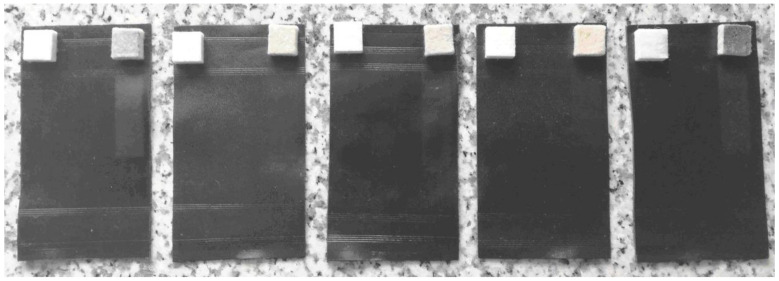
Images of the leather specimens after rubbing test with dry (left) and wet (right) felt. (Samples from left to right: DS 90/10, DS 80/20, DS 75/25, DS 60/40, DS 50/50).

The results showed that with the increase of MMA shell ratio the flexing endurance seemed to decrease as well. For instance, the leather coated with DS 50/50 showed large grain cracks with damaged areas whereas the leather with DS 90/10 showed no visible damage. This result was expected, as the increase of MMA shell ratio also enhances the brittleness of the final film resulting in lower flexibility. The rub fastness results of the leathers are also given in Table 4 and leather samples are shown in Figure 5.

After 100 times of rubbing (rubs) under dry conditions, for all samples, no failure was observed; therefore, the test was kept till 500 rubs. As it can be seen, all leathers finished with core–shell latexes as binders showed good rub fastness values after 500 rubs. The rubbing test was also performed using wet felt for 10 and 20 times. The results showed that even after ×10 rubbing action considerable damage on the leather finish and coloring of felt was observed for leathers processed with DS 50/50 and DS 90/10. Even though, the leathers processed with the other core–shell ratios gave better performance to wet rubbing, the best considered results were obtained for leather DS 60/40. Usually, the performance of a finish layer to wet rubbing action improves with increasing film hardness. Therefore, a proportional enhancement for wet rubbing fastness was observed with the increase of hard segment to an extent. In the case of the sample DS 50/50, the polymeric layer might be too hard so that the adhesion of finishing layer was significantly reduced, leading to incompatibility with the substrate and the fastness values decreased accordingly.

**Figure 6:**
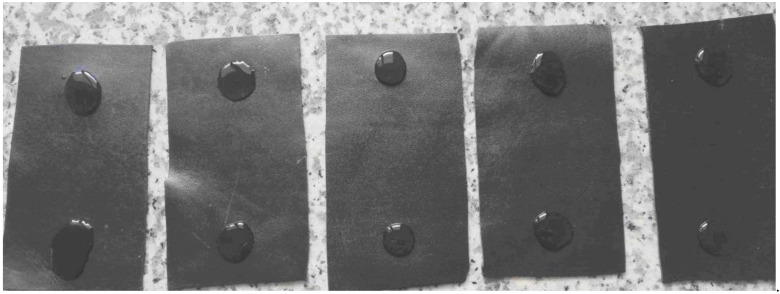
The images of the leather specimens during water spotting test after 30 min. (Samples from left to right: DS 90/10, DS 80/20, DS 75/25, DS 60/40, DS 50/50).

Water spotting test was also performed to assess the behavior of leathers to water. During the test, two drops of water were placed on the leather specimens (Figure 6). After 30 min the residual water of one spot was gently removed by filter paper and physical effects were observed. For the other spot leather was left to stand 16 h to assess the change in color with the grey scale. After 30 min, it was observed that all the water drops remained on the leather surface, and no physical damage or change was observed after removing the drop. Similarly, no change in color was observed on the leathers after 16 h, showing that all the leathers exhibited good performance to the water spotting.

## 4. Conclusions

Composite polyacrylate latex series based on poly (ethylacrylate–*co*–itaconic acid/polymethyl metacrylate with different soft core/hard shell ratios (from 90/10 to 50/50) were synthesized successfully via two stage seeded emulsion polymerization technique. Most of the latexes had good stability with low average particle size and narrow size distributions. The increase of the hard-shell ratio did not have a significant effect on particle size and distributions; however, at high shell ratios (DS 50/50) the stability was slightly affected, as some coagulum was observed at the end of the reaction. The two different polymer phases of the latex were confirmed by thermal and mechanical analyses. The obtained latexes were further used as binders in leather finishing application and the effect of phase ratios on coating performance was evidenced with physical tests performed on leathers. The evaluation of overall results showed that the increase of hard-shell ratios had positive effect on mechanical and especially wet performance of the coating. On the other hand, the flexibility of the coating was decreased as expected, which is important for coating of soft and flexible surface materials. Moreover, a further increase in the ratio of hard segments such as DS 50/50 makes the final film too brittle for flexible coatings, causing reduction in performance levels. The use of itaconic acid moiety within the monomer composition seemed to increase the stability of the emulsions, as they most latexes were obtained coagulum free. Moreover, itaconic acid with double carboxyl functional groups also increases hydrogen bonding capacity of the coating, thus promoting the adhesion with to the substrate, as very good results obtained for rubbing fastness. It can be concluded that the increase of the hard-shell phase ratio has positive effects on coating performance levels up to a certain ratio and the composition should be adjusted carefully according to the substrate and desired coating properties.

## Figures and Tables

**Table 1:** Experimental set-up and reaction parameters for copolymer emulsion.

Sample Code			Experimental Set-Up			Results	
	Seed (g) (EA + IA)		Core (g) (EA)	Shell (g) (MMA)	APS (g)	Solid wt %	Conversion %
DS 90/10	3.5	1	36.00	4.50	0.35	25	99
DS 80/20	3.5	1	31.50	9.00	0.35	25	99
DS 75/25	3.5	1	29.25	11.25	0.35	25	99
DS 60/40	3.5	1	22.50	18.00	0.35	25	99
DS 50/50	3.5	1	18.00	22.50	0.35	25	99

**Table 2:** The finishing formulation applied on the leathers.

Components	Application Steps		Descriptions
	Basecoat (I) (Parts Per Unit)	Topcoat (II) (Parts Per Unit)	
Water	35	20	Spray I × 3 times
Pigment	10		Hot plate 90 °C/100 bar
Wax	5		
Core–shell Latex	30		Spray I × 2 times
Polyurethane Emulsion	5		Hot plate 90 °C/70 bar
Casein Emulsion	5		
Isopropyl alcohol	0.5		
Aqueous NC Lacquer		10	Spray II × 1 time

**Table 3:** Average particle size diameter and polydispersity index values of the latexes.

Sample Code	Size (nm)	Polydispersity Index (PDI)
DS 90/10	113.9 ± 2.35	0.0570 ± 0.008
DS 80/20	119.7 ± 2.37	0.1280 ± 0.013
DS 75/25	83.1 ± 5.56	0.0148 ± 0.005
DS 60/40	140.4 ± 4.53	0.0021 ± 0.0011
DS 50/50	173.3 ± 2.75	0.0580 ± 0.006

**Table 4:** The physical test results applied on finished leathers.

Leather Sample.	Flexing Endurance (×200,000)	Fastness Level—Grey Scale								
		100 Rubs (Dry)		500 Rubs (Dry)		10 Rubs (Wet)		25 Rubs (Wet)		Water Spotting (16 h)
		Leather	Felt	Leather	Felt	Leather	Felt	Leather	Felt	Leather
DS 50/50	Large Grain Cracks	5	5	4/5	4	2	1	1	1	5
DS 60/40	Fine Grain Cracks	5	5	5	5	4/5	5	4	4	5
DS 75/25	Fine Grain Cracks	5	5	4/5	5	4/5	4/5	3	2	5
DS 80/20	Fine Grain Cracks	5	5	4/5	5	4/5	4	3	2/3	5
DS 90/10	Excellent	5	5	5	5	1	3	1	1/2	5

## Data Availability

The data presented in this study are available on request from the corresponding author.
